# Central retinal artery occlusion in a patient with aortic valve papillary fibroelastoma: a case report

**DOI:** 10.1093/ehjcr/ytag048

**Published:** 2026-01-25

**Authors:** Fabian Kerwagen, Antony William, Nodir Madrahimov, Sabrina Strobel, Nils Petri

**Affiliations:** Department of Clinical Research and Epidemiology, Comprehensive Heart Failure Center, University Hospital Würzburg, Würzburg, Am Schwarzenberg 15, 97078 Würzburg, Germany; Department of Internal Medicine I, University Hospital Würzburg, Würzburg, Oberdürrbacher Straße 6, 97080 Würzburg, Germany; Department of Ophthalmology, University Hospital Würzburg, Würzburg, Josef-Schneider-Straße 11, 97080 Würzburg, Germany; Department of Thoracic and Cardiovascular Surgery, University Hospital Würzburg, Würzburg, Oberdürrbacher Straße 6, 97080 Würzburg, Germany; Institute of Pathology, Julius-Maximilian-University of Wuerzburg, Würzburg, Josef-Schneider-Straße 2, 97080 Würzburg, Germany; Department of Clinical Research and Epidemiology, Comprehensive Heart Failure Center, University Hospital Würzburg, Würzburg, Am Schwarzenberg 15, 97078 Würzburg, Germany; Department of Internal Medicine I, University Hospital Würzburg, Würzburg, Oberdürrbacher Straße 6, 97080 Würzburg, Germany

**Keywords:** Papillary fibroelastoma, Central retinal artery occlusion, Embolic stroke, Echocardiography, Cardiac surgery, Case report

## Abstract

**Background:**

Cardiac papillary fibroelastoma are rare benign tumours of the endocardium, often arising from heart valves. Despite their benign histology, papillary fibroelastoma carry high embolic potential and can lead to ischaemic events. In contrast to stroke, ocular embolization including central retinal artery occlusion is a rather rare first manifestation.

**Case summary:**

We report a 61-year-old female with sudden painless monocular vision loss due to central retinal artery occlusion. Initial stroke and cardiovascular work-up revealed a very small floating mass (0.6 × 0.4 × 0.2 cm) on the aortic valve by transesophageal echocardiography. The patient underwent successful surgical excision, with histopathology confirming papillary fibroelastoma.

**Discussion:**

Central retinal artery occlusion as a primary manifestation of papillary fibroelastoma highlights the embolic potential of these tumours. Multimodality imaging, especially transesophageal echocardiography, is crucial in diagnosis. Even very small papillary fibroelastoma may cause devastating embolic events such as irreversible monocular blindness. Surgical excision is the treatment of choice to prevent recurrence.

Learning points

- Papillary fibroelastoma is a benign cardiac tumour with high embolic risk, and may first present with ocular ischaemia.
- Central retinal artery occlusion should trigger an urgent search for cardioembolic sources, including rare tumours.
- Transesophageal echocardiography is the most sensitive diagnostic approach for detecting papillary fibroelastoma.
- Surgical excision is recommended in symptomatic cases to prevent recurrent embolic complications.

## Introduction

Papillary fibroelastoma is a rare cardiac tumour, with an incidence of 0.038% among patients undergoing echocardiography,^1^ and accounting for about 11.5% of all primary cardiac tumours.^2,3^ While often asymptomatic, papillary fibroelastomas are strongly associated with embolic complications, particularly ischaemic stroke and transient ischaemic attack.^4^ Ocular arterial embolism as a first manifestation is an extremely rare but clinically significant occurrence.

## Summary figure

**Figure:**
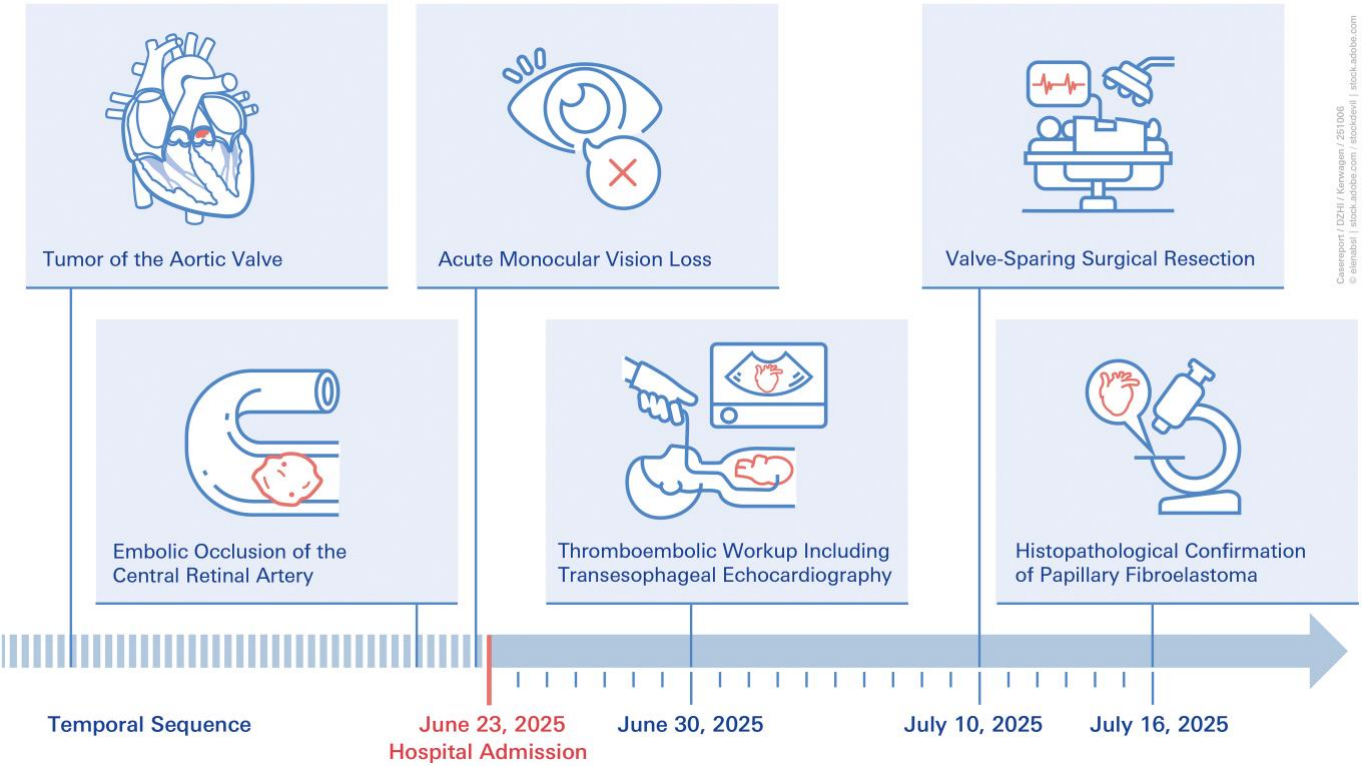


## Case presentation

**Figure 1:**
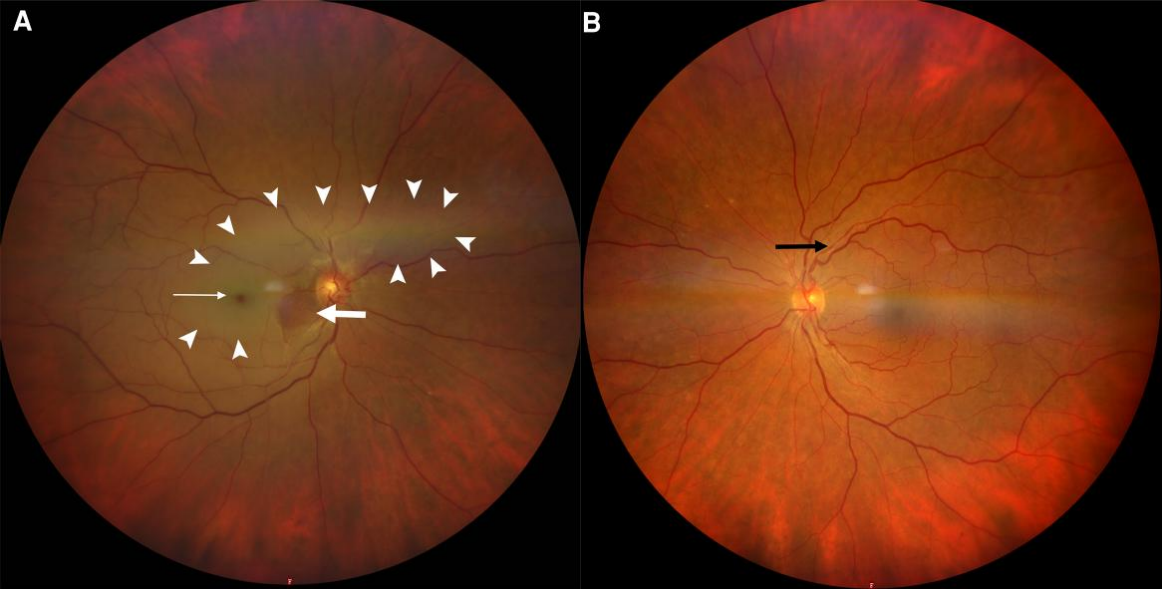
Findings of fundoscopy. Panel A shows the fundus of the patient’s right eye. There is retinal pallor (white arrowheads, indicating ischaemic inner retinal tissue), a cherry red spot (white thin arrow, reflecting preserved choroidal perfusion at the fovea), and a small area of normally perfused retina inferior to the optic disc due to a patent cilioretinal artery (white block arrow). Panel B shows the fundus of the contralateral, healthy (left) eye with a normally perfused retina and arteriolar narrowing (black arrow, a sign of chronic hypertensive vascular changes).

**Figure 2:**
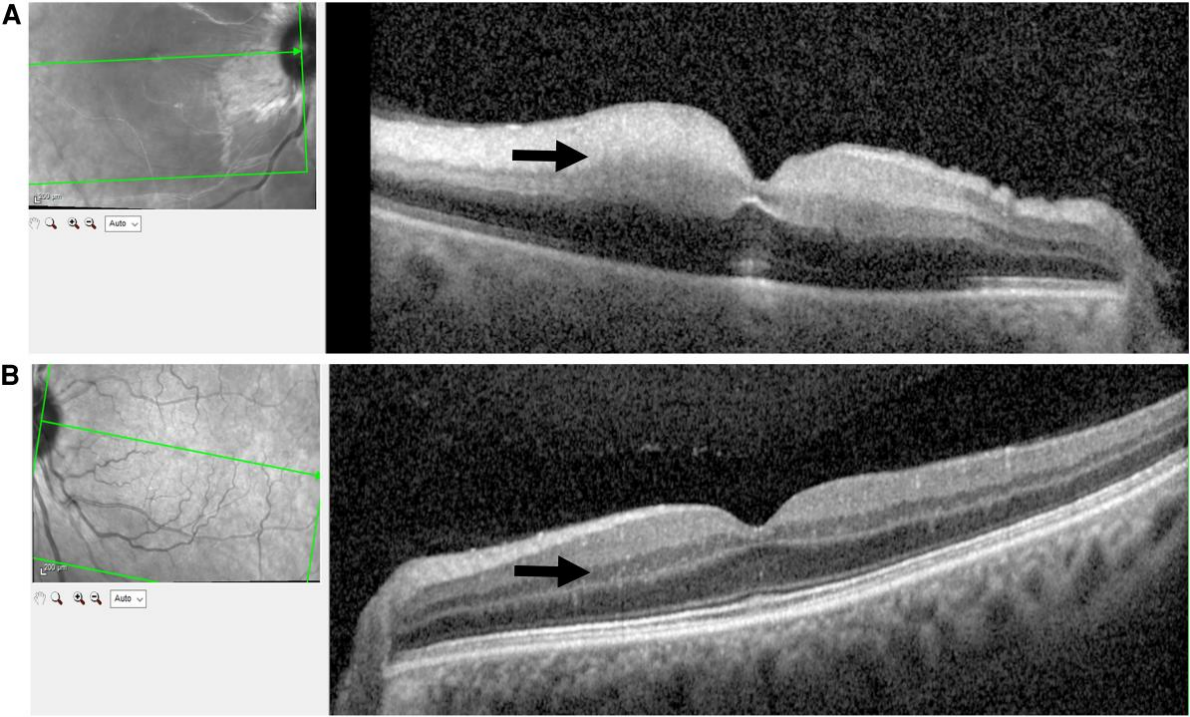
Findings of optical coherence tomography. Panel A shows thickening of the inner retinal layers (arrow) due to cytotoxic oedema. Panel B shows the healthy retina of the contralateral eye with normal retinal layers (arrow).

A 61-year-old female with a history of arterial hypertension was referred to the emergency department with acute, painless loss of vision in the right eye on 23 June 2025 (*Summary Figure*). The patient reported that she awoke at 5:30 a.m. and immediately noticed the visual loss. She initially consulted a community-based ophthalmologist, who subsequently referred her to our clinic, where she presented a few hours later on the same day. Ophthalmological examination confirmed central retinal artery occlusion through the findings by fundoscopy (*Figure 1*) and optical coherence tomography (*Figure 2*). Neurological examination showed no additional focal deficits.

Routine blood work, inflammatory markers, and coagulation profile were normal. Given the embolic presentation, a cardioembolic work-up was initiated. Electrocardiogram (ECG) and 24-hour long-term ECG showed sinus rhythm. Carotid Doppler sonography showed no evidence of haemodynamically relevant stenosis.

**Figure 3:**
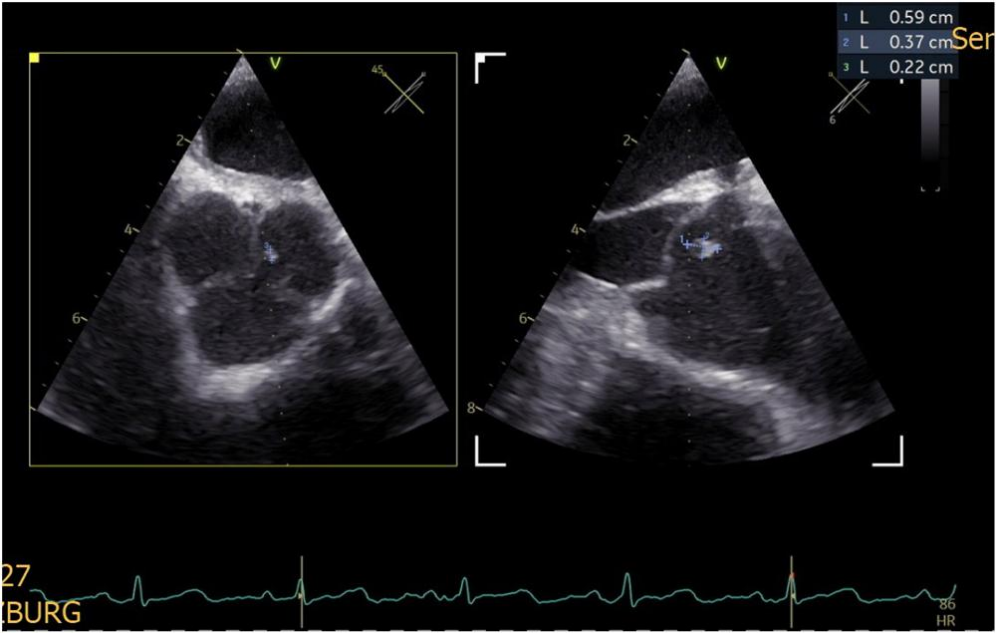
Transesophageal echocardiography demonstrating a small, mobile mass attached to the left coronary cusp of the aortic valve, consistent with papillary fibroelastoma. A corresponding video illustrating this finding is available in the Supplementary material.

**Figure 4:**
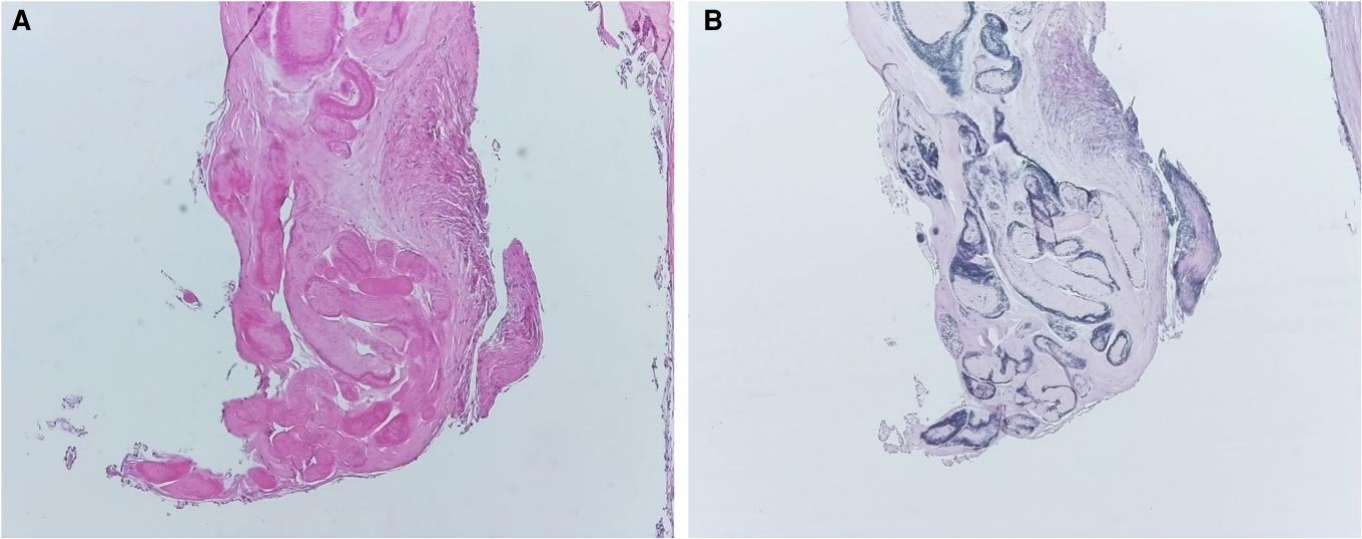
Histopathological sections of the resected papillary fibroelastoma. (*A*) Haematoxylin–eosin staining shows the fibroelastic tissue with blue-stained nuclei and pink-stained cytoplasm and collagenous structures. (*B*) Elastika–van-Gieson staining highlights the fibroelastic architecture, with collagen fibres in pale red, elastic fibres in black-violet, nuclei in black-blue/brown, and cytoplasm in yellow. Both stainings demonstrate the characteristic morphology of a papillary fibroelastoma.

Seven days after hospital admission, transesophageal echocardiography revealed a small (0.6 × 0.4 × 0.2 cm) floating mass attached to the left coronary cusp of the aortic valve (*Figure 3* and Video in the Supplement). While the lesion was also visible on transthoracic echocardiography, it was not detected by computed tomography (CT). After ruling out significant coronary artery stenosis by CT, the patient underwent surgical resection of the tumour under cardiopulmonary bypass on the 10 of July 2025. The mass was completely excised, and valve preservation was achieved. Six days after resection, histopathological examination confirmed the diagnosis of papillary fibroelastoma (*Figure 4*). Post-operatively, the patient remained stable. Ophthalmological outcome was characterized by persistent severe vision loss in the right eye, consistent with irreversible central retinal artery occlusion.

## Discussion

Papillary fibroelastoma, though histologically benign, is associated with embolic complications. In a recently published comprehensive systematic review including 161 patients with papillary fibroelastoma, ischaemic stroke occurred in 68% and transient ischaemic attack in 32% of cases. Visual symptoms were described in 17% of these patients.^4^ However, isolated central retinal artery occlusion as the initial manifestation is exceedingly rare. For instance, Lopez-Sanchez *et al*. reported central retinal artery occlusion as the initial manifestation of an aortic valve papillary fibroelastoma in a young 32-year-old male patient. Similar to our case, the diagnosis was established by echocardiography and confirmed histologically after surgical resection, while visual outcome remained poor despite timely intervention.^5^ Our case adds to this limited body of evidence by highlighting that central retinal artery occlusion may also occur in older patients and may be associated with very small, highly mobile lesions. Together, these observations underscore that papillary fibroelastoma, although rare, should be considered in the differential diagnosis of unexplained central retinal artery occlusion, particularly when no alternative embolic source is identified. In general, diagnosis relies on imaging. While other diagnostic approaches including computed tomography or transthoracic echocardiography may miss small, mobile tumours, transesophageal echocardiography provides a higher sensitivity.

The differential diagnosis of small valvular lesions includes Lambl’s excrescences (valvular strands), which are thin, filamentous degenerative strands typically located along valve closure lines.^6–8^ In contrast to papillary fibroelastoma, which represents a true cardiac tumour with frond-like morphology and increased embolic potential, Lambl’s excrescences are far more common and often detected incidentally without clinical symptoms. Some studies have reported an association between valvular strands and embolic events,^6–8^ but they do not appear to affect overall mortality.^6^ Distinguishing between these entities is therefore clinically relevant, as management strategies differ substantially, ranging from conservative surveillance in most cases of Lambl’s excrescences to surgical excision in symptomatic or high-risk papillary fibroelastoma (*Table 1*).

**Table 1:** Key differences between papillary fibroelastoma and Lambl’s excrescences (valvular strands)

Feature	Papillary fibroelastoma	Lambl’s excrescences (valvular strands)
Nature	True benign cardiac tumour	Degenerative, non-neoplastic
Morphology	Pedunculated, frond-like, highly mobile mass	Thin, thread-like filamentous strands located along valve closure lines
Typical size	Several millimetres, often up to or >1 cm	Usually very thin (<2 mm), filiform projections
Embolic risk	Increased/clinically relevant	Associated with embolic events, but clinical relevance remains controversial
Management	Surgical excision in symptomatic or high-risk cases	Conservative management in most cases

Due to the rarity of papillary fibroelastoma, international guidelines regarding management are lacking. Surgical excision is generally recommended in symptomatic patients and in those with left-sided, mobile lesions because of their increased embolic potential. In contrast, a conservative strategy with clinical and echocardiographic surveillance may be considered in asymptomatic patients with small, non-mobile lesions and without prior embolic events. However, in asymptomatic or incidentally detected papillary fibroelastoma with high-risk features such as marked mobility, pedunculated morphology, or prior embolic events, preventive surgery might be advocated. In the present case, the occurrence of a severe embolic complication, the left-sided location on the aortic valve, and the highly mobile nature of the lesion provided a clear indication for surgical removal. In the systematic review, 95% of patients underwent excision, with valve-sparing shave resection sufficient in most cases. Post-operative recurrence is rare but has been described.

Our patient exemplifies how central retinal artery occlusion, although rare, can be the first manifestation of cardiac papillary fibroelastoma. Prompt recognition and surgical management may prevent further embolic complications. Although stroke is much more common, central retinal artery occlusion should also prompt comprehensive cardioembolic work-up and cardiac tumours should also be considered potential cause.

## Conclusion

We report a rare case of papillary fibroelastoma presenting as isolated central retinal artery occlusion. Central retinal artery occlusion should be recognized as a possible manifestation of cardiac tumours, and a thorough cardiac evaluation including transesophageal echocardiography is warranted. Surgical excision remains the treatment of choice to prevent recurrence of embolic events.

## Lead author biography


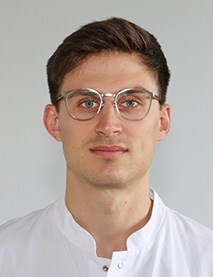


Fabian Kerwagen, MD, MPH, is a cardiology resident in the Department of Internal Medicine I at the University Hospital Würzburg, Germany, and a research associate at the Comprehensive Heart Failure Center in Würzburg. His research focuses on digital health innovations, remote patient management, and translational strategies to improve heart failure care. He contributes to national and European research consortia on digital health and vocal biomarkers. Dr Kerwagen is actively involved in academic societies and currently serves as the National Ambassador of the Young Heart Failure Association for Germany, dedicated to advancing evidence-based cardiovascular medicine through clinical education and interdisciplinary research.

## Supplementary Material

ytag048_Supplementary_Data

## Data Availability

Data supporting this report are available from the corresponding author upon reasonable request.
